# Automated left atrial time-resolved segmentation in MRI long-axis cine images using active contours

**DOI:** 10.1186/s12880-021-00630-3

**Published:** 2021-06-19

**Authors:** Ricardo A. Gonzales, Felicia Seemann, Jérôme Lamy, Per M. Arvidsson, Einar Heiberg, Victor Murray, Dana C. Peters

**Affiliations:** 1 Department of Radiology and Biomedical Imaging, Yale School of Medicine, Yale University, New Haven, Connecticut United States of America; 2 Department of Electrical Engineering, Universidad de Ingeniería y Tecnología, Lima, Peru; 3 Department of Clinical Physiology, Lund University, Skåne University Hospital, Lund, Sweden; 4 Department of Biomedical Engineering, Lund University, Lund, Sweden; 5 Wallenberg Center for Molecular Medicine, Lund University, Lund, Sweden; 6 John A. Paulson School of Engineering and Applied Sciences, Harvard University, Cambridge, Massachusetts United States of America; 7 Department of Electrical and Computer Engineering, University of New Mexico, Albuquerque, New Mexico United States of America

**Keywords:** Active contours, Cardiovascular imaging, Magnetic resonance imaging, Left atrium, Segmentation

## Abstract

**Background:**

Segmentation of the left atrium (LA) is required to evaluate atrial size and function, which are important imaging biomarkers for a wide range of cardiovascular conditions, such as atrial fibrillation, stroke, and diastolic dysfunction. LA segmentations are currently being performed manually, which is time-consuming and observer-dependent.

**Methods:**

This study presents an automated image processing algorithm for time-resolved LA segmentation in cardiac magnetic resonance imaging (MRI) long-axis cine images of the 2-chamber (2ch) and 4-chamber (4ch) views using active contours. The proposed algorithm combines mitral valve tracking, automated threshold calculation, edge detection on a radially resampled image, edge tracking based on Dijkstra’s algorithm, and post-processing involving smoothing and interpolation. The algorithm was evaluated in 37 patients diagnosed mainly with paroxysmal atrial fibrillation. Segmentation accuracy was assessed using the Dice similarity coefficient (DSC) and Hausdorff distance (HD), with manual segmentations in all time frames as the reference standard. For inter-observer variability analysis, a second observer performed manual segmentations at end-diastole and end-systole on all subjects.

**Results:**

The proposed automated method achieved high performance in segmenting the LA in long-axis cine sequences, with a DSC of 0.96 for 2ch and 0.95 for 4ch, and an HD of 5.5 mm for 2ch and 6.4 mm for 4ch. The manual inter-observer variability analysis had an average DSC of 0.95 and an average HD of 4.9 mm.

**Conclusion:**

The proposed automated method achieved performance on par with human experts analyzing MRI images for evaluation of atrial size and function.

**Video Abstract**

**Supplementary Information:**

The online version contains supplementary material available at 10.1186/s12880-021-00630-3.

## Introduction

In the United States, the lifetime risk of developing atrial fibrillation (AF) is 1 in 4, in people over 40 years old [1], and 6 million Americans are living with left-ventricle (LV) heart failure [2]. AF is an abnormal heart rhythm characterized by a rapid and irregular heartbeat, produced by ectopic beats originating from the left atrium (LA) [3]. LV heart failure, with or without reduced ejection fraction (EF), is a condition in which the heart fails to produce adequate blood flow to the body. The evaluation of AF, stroke risk, heart failure, and other cardiomyopathies is potentially enhanced by assessment of atrial volumes, function, and strain. For example, larger LA volumes and lower strain and LA EF were independent predictors of AF development in recent studies [4, 5], including sub-clinical AF [6]. LA function may improve the diagnostic accuracy and prognostic value of diastolic dysfunction in magnetic resonance imaging (MRI) [7]. LA evaluation plays an important role in imaging of AF, stroke risk related to AF, and diastolic dysfunction evaluation [8]. Despite its importance, values for LA volumes and function are not often reported clinically, due in part to the lack of available image processing tools for the LA.

Segmentation of the LA is required for evaluation of LA size and function [9], which are relevant diagnostic and prognostic imaging biomarkers for diverse cardiovascular conditions. LA strain evaluation is a more recent and potentially superior biomarker of cardiac disease, compared to LA size and function. Evaluation for all of these measures requires LA segmentation [10–13], and for strain the segmentation must be highly accurate and include all time-frames.

Cardiac MRI is considered the reference standard for cardiac volume assessment, offering accurate evaluation of LV and LA structure and function [14]. Cardiac MRI is routinely used to acquire time-resolved 2-chamber (2ch) and 4-chamber (4ch) long-axis cines of the heart (multiple 2D slices), providing high-contrast cardiac images at multiple points in the cardiac cycle. From these cine data, LA volumes can be measured, using the bi-plane method [15], and function can be estimated, based on the changes in LA volume over the heartbeat.

Volumes and strain during the entire cardiac cycle are essential, both for calculating volume-flow rates and strain rates, which require time derivatives, and for evaluation during multiple phases of the cardiac cycle, which include reservoir and conduit phase (filling in LV systole, and passive emptying in early diastole) and the active emptying phase. For the most part, LA segmentations are currently being performed manually, which is time-consuming and observer-dependent, underscoring the need for automated segmentation algorithms.

Advances in automated heart segmentation have primarily focused on the LV [16], secondarily on the right ventricle [17], whereas an automated segmentation method for the LA has yet to be routinely used in clinical practice [16] for several reasons. The LA chamber is typically smaller than the LV, with a thin myocardial border and a very variable shape across subjects. Boundaries are not clearly defined, primarily (i) the boundary between the LA cavity and pulmonary vein (PV) ostia, (ii) between the LA cavity and the left atrial appendage (LAA), and (iii) the mitral valve (MV) boundary, which separates the LA from the LV. Moreover, the MV has a dynamic motion throughout the cardiac cycle and is almost invisible in diastole.

Most LA segmentation methods are focused on whole heart images [18], with only a few methods developed for LA cine. Regarding these, one method uses feature tracking to propagate LA contours, once manually initialized on end-diastolic and/or end-systolic frames [10, 19], but this still demands user interaction. Machine learning techniques have been used to segment the LA in long-axis cine images, integrating a modified U-net based on deep convolutional neural networks with the unscented Kalman filter to impose temporal consistency in 2, 3 and 4-chamber views [20]. Another convolutional neural network architecture with a VGG-16 framework was proposed to predict a pixel-wise label map for the LA [21], but it was only trained on end-diastolic and end-systolic frames and with healthy volunteers. Regarding segmentation performance, the proposed methods provided high accuracy. However, from visual inspection it is clear that none of these methods provided a smooth delineation of the PV and LAA and a flat cut across the dynamic MV, nor did they present clinically validated, derived LA parameters (i.e., volumes, EFs, or strains) from the generated contours with validation against manual segmentation. Therefore, an automated method capable of segmenting all cine frames, with high accuracy, including validation of clinically utilized parameters, is still needed.

In this paper, we propose an automated LA time-resolved segmentation method for long-axis cine images of the 2ch and 4ch via active contours. The method combines MV tracking (fully or semi-automated), automated threshold calculation, edge detection on a radially resampled image, cost image optimization based on a Dijkstra algorithm and post-processing involving smoothing and interpolation. The resulting volumes and functional parameters are then compared to manual segmentation.

**Fig. 1:**
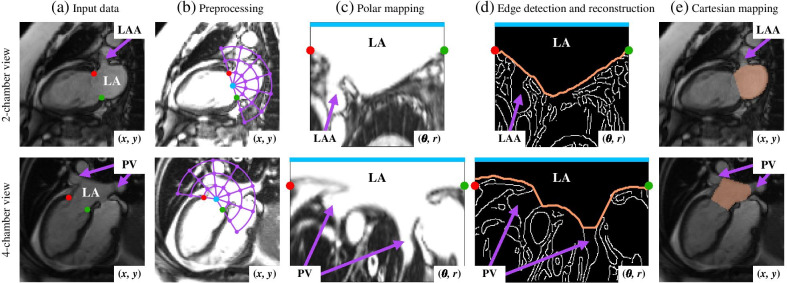
Workflow to automatically segment the left atrium (LA) of a subject in 2-chamber (2ch) and 4-chamber (4ch) views. The left atrial appendage (LAA) in 2ch and the pulmonary veins (PVs) in 4ch are marked in purple. **a** Input images, with mitral valve (MV) annotations, the red and green points respectively correspond to the anterior and inferior points for 2ch or the lateral and septal points for 4ch. **b** Data preprocessing uses an automatically calculated threshold value and assigns a signal above the threshold to the threshold value. The polar mapping is performed using a reference point, shown in blue (defined as the MV center), and a polar grid, in purple, within the Cartesian plane (*x*, *y*). **c** Resultant image after polar mapping $(\theta ,r)$ of the LA constrained by the MV points. The blue line represents $r=0$ for all the angular range, which spans 180$^{\circ }$ for 2ch and 233$^{\circ }$ for 4ch. **d** The images in **c** are subjected to Canny edge detection followed by active contours, resulting in a final contour shown in orange. **e** The contours are remapped onto a Cartesian grid

**Fig. 2:**
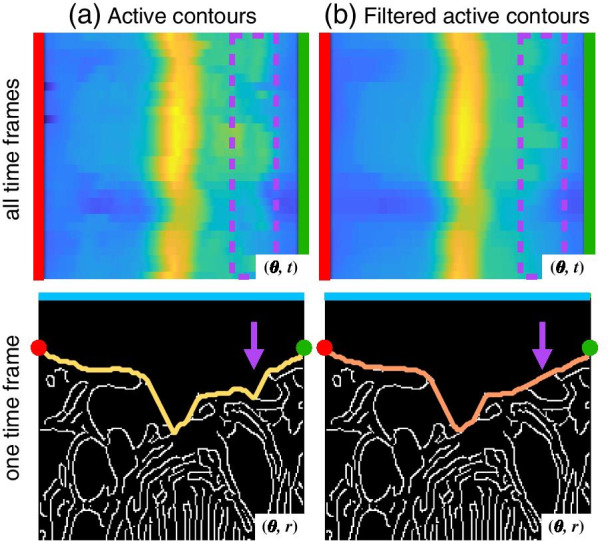
Post-processing of reconstructed edges within the proposed workflow of a subject in 2-chamber view, displayed in heat maps $F(\theta ,t)$ and polar grids with their corresponding active contours $f(\theta ,r)$. The heat maps (firs row) display the distance from the valve central point to the detected active contour, for every angle and every time frame, while the polar grids (second row) display the edge location in a time frame, similar to Fig. 1d. The mitral valve annotations are also displayed in red and green. **a** The detected active contours obtained with the proposed optimization problem before filtering. **b** With post-processing, the heat map is smoother and the false peaks were removed, marked in purple

**Table 1:** Automated segmentation accuracy ($\mathrm{n}=37\times 30$) of 2-chamber and 4-chamber view evaluated in Dice similarity coefficient (DSC), mean contour distance (MCD) and Hausdorff distance (HD)

	DSC	MCD (mm)	HD (mm)
2-chamber set	0.96 ± 0.028	1.2 ± 0.53	5.5 ± 3.0
4-chamber set	0.95 ± 0.042	1.3 ± 0.59	6.4 ± 3.7

The mean ± standard deviation are reported

**Table 2:** Manual inter-observer agreement ($\mathrm{n}=37\times 2$) of 2-chamber and 4-chamber view evaluated in Dice similarity coefficient (DSC), mean contour distance (MCD) and Hausdorff distance (HD)

	DSC	MCD (mm)	HD (mm)
2-chamber set	0.95 ± 0.046	1.4 ± 0.68	5.0 ± 2.6
4-chamber set	0.95 ± 0.038	1.2 ± 0.46	4.8 ± 2.5

The mean ± standard deviation are reported

## Methodology

We propose an automated LA segmentation based on active contours on a polar grid. The input data passed through four key steps: (i) preprocessing, (ii) polar mapping, (iii) edge detection and reconstruction, and (iv) Cartesian mapping. Figure 1 depicts the segmentation workflow in both views from a subject.

### Input data

The LA segmentation method used long-axis cine images of the 2ch and 4ch views, acquired in a breath-hold with a balanced steady-state free precession sequence. Each cardiac cycle was composed of 30 images, representing time frames. Each image had an average spatial resolution of $2 \times 2 \times 8$ $\hbox {mm}^3$, and the MRI sequence acquisition parameters were a repetition time of 3 ms, an echo time of 1.5 ms, and a flip angle of 60$^{\circ }$.

### Preprocessing

The MV insertion points were obtained in a semi-automated manner, using feature tracking [22], and in a fully-automated manner, using residual neural networks [23], for comparison. The first approach was based on template tracking by normalized cross-correlation and a priori information by principal component analysis, and required a manual initialization of the MV points at end-diastole. The second approach employed a deep learning-based landmark annotation with no user interaction, whose sensitivity was tested (see Reproducibility due to variability in MV point placement section). These points define the MV plane, which separates the LA from the LV. Figure 1a illustrates the placement of the MV points. For the case of 2ch, the red and green points correspond to the anterior and inferior points, respectively, whereas for the case of 4ch, they correspond to the lateral and septal points.

To avoid false edge detection within the blood pool, all blood pool pixels with similar intensity were assigned under a single threshold value. To implement this threshold, the input image was considered as a Gaussian mixture of two-component pixel intensities, corresponding to sections of low intensity (muscle tissue), and high intensity (blood tissue). The Gaussian mixture parameters were obtained by an adapted Expectation-Maximization algorithm [24]. The threshold value was defined as the mean of the highest Gaussian distribution. Figure 1b shows the preprocessing step on the same subject, in which all pixels above the threshold were set to the threshold value.

### Polar mapping

The cine images were mapped from a Cartesian grid (*x*, *y*) to a polar grid $(\theta ,r)$. Under this constraint, due to the geometric LA shape, other borders within the LA, related to the PV ostia and LAA, would be superimposed by the LA myocardium and thereby excluded using active contours.

The MV points were used to delimit the dynamic mitral valve and to initialize the polar mapping step in each frame. The reference point, illustrated in blue in Fig. 1b, was set in the middle of the mitral valve axis defined by the MV annotations. The radial range $N_r$ covered twice the mitral valve length, whereas the angular range $N_\theta$ was uniformly limited by the MV points, meaning it mapped from anterior to inferior point for 2ch or from lateral to septal point for 4ch, in other words, following the same color bar of Fig. 1, from red to green point. Due to the MV points’ dynamic behavior along the cardiac cycle, the reference point was updated for every time frame.

This task was performed in both views with a difference in the reference point position. This point was set exactly in the middle of the MV annotations for 2ch. However, for 4ch, the point was perpendicularly moved above the mitral valve plane for a quarter of its length, as illustrated in Fig. 1b. This change was due to the prolonged and curvilinear LA shape on this view, which was captured by this adjustment. The resampled LA shape in both cases resembled a “V” shape, as shown in Fig. 1c.

The polar map was generated with sampling intervals $\Delta r$ of 0.25 pixels and $\Delta \theta$ of $1^{\circ }$, to resample the LA region from Cartesian coordinates, for each frame. The radial range varied from 150 to 200 rows depending on the mitral valve length, whereas the angular range was fixed to 180 or 230 columns, for 2ch and 4ch, respectively, as a result of the different reference point positions. The resultant image was interpolated with a bilinear resampling and its resolution was controlled by the sampling intervals.

### Edge detection and reconstruction

A Canny edge detection technique [25] was used to robustly find the edges in each polar image. This resulted in a 2D matrix, considered to be the cost image $I_\text {c}(i,j)$, with white pixels representing detected edges, with the MV points and reference line in polar coordinates as shown in Fig. 1d.

The presumed LA edge was found from $I_\text {c}(i,j)$ using an optimization algorithm based on active contours, in each polar image. This active contour *f* was modeled as a physical string and defined as the minimization of its internal energy, $E_\text {int}(f)$, and the energy of its position on the image, $E_\text {im}(f,I_\text {c})$, by

1 $$\begin{aligned} \min _{f}\left\{ E_\text {int}(f)+E_\text {im}(f,I_\text {c})\right\} . \end{aligned}$$

The use of active contours facilitated the manipulation of physical behavior with the tuning parameters elasticity, $\alpha$, and rigidity, $\beta$. The elasticity controlled the amount of stretch and penalized changes from point to point in the contour, while the rigidity controlled the amount of curvature [26]. These tuning parameters defined the internal energy, first part of Eq. (1), as

2 $$\begin{aligned} E_\text {int}(f)=\frac{1}{2}\int \left( {\alpha \cdot f'(s)^2+\beta \cdot f''(s)^2)}\right) ds. \end{aligned}$$

An optimization problem positioned in a Cartesian grid in this case would yield local optimums, i.e., non-convex problem, which may not be the best solution. The polar mapping enabled a convex problem restricting the active contour to a proper discrete function of its own $f:\text {N}^1\rightarrow \text {N}^1$, restricted to only pass through each column ($\theta$) in the grid once. With the reformulation of the optimization problem, described and solved with dynamic programming in [27], $E_\text {int}(f)$, or Eq. (2), was discretized with approximate derivatives as

3 $$\begin{aligned} E_\text {int}(f) & = \sum _{i=1}^{N_r-1} \alpha (f_i-f_{i+1})^2 \\&\quad + \sum _{i=2}^{N_r-1} \beta (-f_{i-1}+2 f_i-f_{i+1})^2, \end{aligned}$$

where $N_r$ is the radial range previously described, and the index of *f* belongs to $1\dots N_\theta$, being $N_\theta$ the angular range, whereas $E_\text {im}(f,I_\text {c})$, second part of Eq. (1), was discretized as

4 $$\begin{aligned} E_\text {im}(f,I_\text {c}) & = \sum _{i=1}^{N_r} \sum _{j=f_{i-1}}^{f_i} \frac{|f_i-j|}{|f_{i-1}-f_i|+1} I_\text {c}(i-1,j)\\&\quad + \frac{|j-f_{i-1}|}{|f_{i-1}-f_i|+1} I_\text {c}(i,j). \end{aligned}$$

The discretized line *f* goes from the starting MV point (red) to the ending MV point (green) in the polar image, as illustrated in Fig. 1d. This optimization problem works as follows: (i) the algorithm keeps track of the set of included nodes ($\text {N}$), (ii) in each iteration the set $\text {N}$ is extended with one node until all nodes are included and the algorithm terminates, (iii) nodes are selected so that the total cost from the source node to the included node is minimal, in the same way it is chosen in Dijkstra’s shortest path algorithm, (iv) the cost for including a node is computed from Eq. (3) and (4), (v) for each included node the algorithm also keeps track on the “parent” node, (vi) tentative nodes to be included the set $\text {N}$ are stored in a heap data structure, (vii) the algorithm is initialized with an empty set $\text {N}$, (viii) the optimal path through the image is finally captured by backtracking nodes from end node to start node (see [27] for more details and proof on optimality). This reformulation had the main advantage of maintaining the physical interpretation of treating the contour as a physical string, including its tuning parameters, and optimal solution. As the MV points position sometimes differed from the myocardial edge, the closest edge pixels to the MV points detected in the starting and ending columns were considered to be the contour tips. The $E_\text {int}$ parameters were set as $\alpha = 0.02$ and $\beta = 0.0002$.

A post-processing technique was performed to smooth the surfaces and ensure false peaks were not detected. As the LA in the polar grid resembled a “V” shape, any detected non-predominant peaks in $f(\theta ,r)$ were filtered. All reconstructed contours from a cine image were stacked in a 2D matrix $F(\theta ,t)$, with rows representing the contour in each time frame and with columns representing the radial position of each active contour. This matrix was smoothed with a median filter. Figure 2 shows an example from a 2ch segmentation before and after this post-processing technique, with $F(\theta ,t)$ from the whole cardiac cycle and $f(\theta ,r)$ from a time frame. The result of the post-processing technique shows a smoothed $F(\theta ,t)$ and a $f(\theta ,r)$ without the false peak marked in purple.

### Cartesian mapping

Once each contour was reconstructed and smoothed, it was converted to Cartesian coordinates for the output LA segmentation mask. With the initial reference point and the sampling intervals, the radial magnitude defined the distance from the reference point, and the angular magnitude marked the direction. This mask presented a dynamic cut with the mitral valve, delineated in a D-shaped form, and smooth separations from PV and LAA, as illustrated in Fig. 1e.

### Evaluation

The method was compared against manual segmentation in 37 patients (15 females, age 53 ± 15 years), scanned on a 1.5T clinical MRI (Siemens Healthcare, Erlangen) for diverse cardiovascular indications, mostly paroxysmal AF, who were in sinus rhythm during the MRI cine acquisitions. The subjects were enrolled as part of an IRB-approved chart-review study. This data comprised a total of 1110 images (30 time frames for each patient) for each chamber view separately, with their corresponding manual segmentation performed with a standard operating protocol [28] using the software Segment [29]. For inter-observer variability analysis, a second observer performed manual segmentations at end-diastole and end-systole on all subjects.

Segmentation accuracy was evaluated using the Dice similarity coefficient (DSC) [30], mean contour distance (MCD), and Hausdorff distance (HD) [31]. The DSC was measured as the overlap between two segmentation masks, MCD and HD were measured as the distance error between two segmentation contours with the mean and the maximum values, respectively.

For clinical relevance, LA EDV, LA ESV, LA EF, and LA GLS were calculated from the automated and manual segmentation, as described by others [10, 14, 15]. LA volume (ml) along time *t* was calculated with the bi-plane method as

5 $$\begin{aligned} \text {LA Volume}_t = \frac{16}{3\pi } \frac{\text {Area}^\text {2ch}_t \text {Area}^\text {4ch}_t}{\text {Length}^\text {2ch}_t+\text {Length}^\text {4ch}_t}, \end{aligned}$$

where the areas were obtained with the segmentation contours, and the lengths were calculated on the longitudinal axis as the distance of the perpendicular line measured from the MV center to the superior aspect of the LA. LA EDV (ml) and LA ESV (ml) were measured as the minimum and maximum values of LA volume, respectively, and LA EF (%) as

6 $$\begin{aligned} \text {LA Ejection Fraction} = \frac{\text {LA ESV}-\text {LA EDV}}{\text {LA ESV}}. \end{aligned}$$

LA strain (%), the change in length normalized by the initial $t=1$ length, was calculated using

7 $$\begin{aligned} \text {LA Strain}_t = \frac{\text {Perimeter}^\text {2ch}_t + \text {Perimeter}^\text {4ch}_t}{\text {Perimeter}^\text {2ch}_{t=1} + \text {Perimeter}^\text {4ch}_{t=1}}-1, \end{aligned}$$

where the perimeters were obtained with the segmentation contours excluding the MV. LA GLS (%) was measured as the maximum value of LA strain.

### Reproducibility due to variability in MV point placement

As the position of MV points automatically initialized our LA segmentation process, differences in input points will influence segmentation results. The importance of MV point placement was assessed by adding a variability to the these initialization points. The variability was chosen as the manual inter-observer variability of MV points placement, calculated from 10 subjects to be 1.5 ± 0.7 mm. To study the initialization influence of MV point placement, all the MV points were randomly varied following this inter-observer variability, 100 times. For each simulation, the entire segmentation process was performed, and the segmentation accuracy and the clinical error were assessed against the first automatically obtained segmentations. We also evaluated the LA segmentation accuracy using our automated method for MV point placement [23].

### Comparison to an automated LA segmentation method

The proposed method’s performance was compared against another automated method capable of LA volumes derivation, provided by the commercially available cardiac MR software $\text {CVI}_\text {42}$ (Circle Cardiovascular Imaging, Calgary, Canada). A subset of 26 subjects (1560 images) was used for comparison between manual measures and automated measures of LA EDV and LA ESV. LA EF was manually derived using Eq. (6). LA GLS was not reported by the software. Segmentation masks were not extractable.

### Implementation

The LA segmentation method was developed in MATLAB R2019a (Mathworks, Natick, Massachusetts) and implemented in the medical image analysis software Segment v3.1 R8109 [29] (http://segment.heiberg.se), and is freely available for research purposes.

### Statistical analysis

Clinical metrics comparisons were performed using linear regression analysis, and Bland-Altman plots with manual measurements as a reference, including the bias with limits of agreement ± 1.96 standard deviation [32]. For the analysis, all clinical metrics were obtained for each set, by the observers and the method, and mean error, intra-class correlation coefficient (ICC) with a confidence interval of 95%, correlation value (R), and coefficient of variance (CoV) were calculated.

**Fig. 3:**
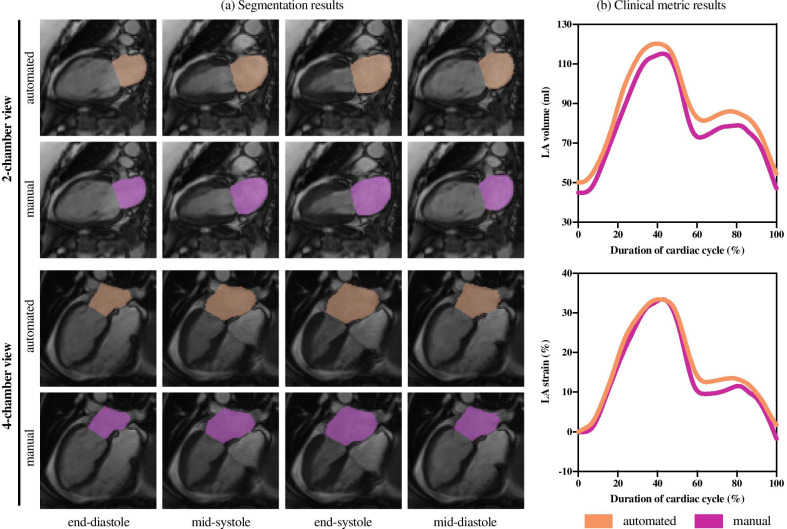
Illustration of the left atrial (LA) segmentation process output for both 2-chamber and 4-chamber views in a subject. **a** Segmentation masks overlayed on input data on 4 different time frames (end-diastole, during systole, end-systole, and during diastole) sequentially represented in columns, in which the top row of each view shows the automated segmentation in orange, whereas the corresponding bottom row shows the manual segmentation in magenta. **b** LA volume and LA strain values vs. time within the cardiac cycle (normalized), obtained by the automated (in orange) and manual (in magenta) measures from both views

**Fig. 4:**
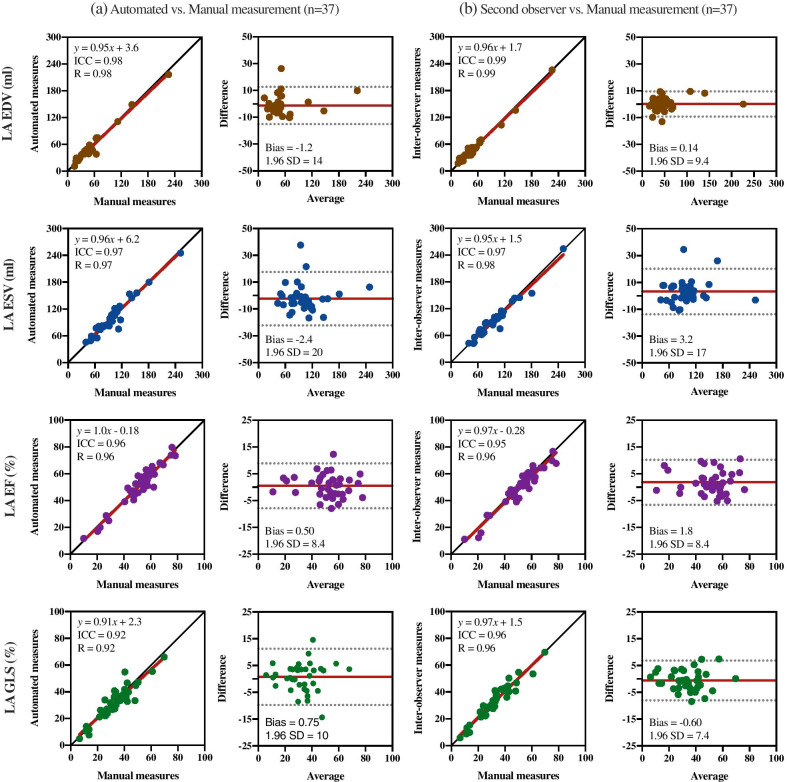
Correlation and bias of left atrial clinical measures between an expert manual segmentation and **a** the automated method, and **b** segmentation by a second observer, on a set of 37 patients. The first column of each analysis shows the regression plots whereas the second shows the Bland-Altman plots of the end-diastolic volume (EDV), in brown, the end-systolic volume (ESV), in blue, the ejection fraction (EF), in purple, and the global longitudinal strain (GLS), in green, distributed in consecutive rows. In each scatter plot the red line denotes the regression line and the black line denotes the identity line, whereas in each Bland-Altman plot, the red line denotes the mean difference (bias) and the two light dotted lines denote ± 1.96 standard deviations from the mean

**Table 3:** Automated clinical metric accuracy ($\mathrm{n}=37$) of left atrial (LA) derived parameters for end-diastolic volume (EDV), end-systolic volume (ESV), ejection fraction (EF) and global longitudinal strain (GLS)

	Manual measures	Auto. measures	Error measures	ICC (95% CI)	R	CoV (%)
LA EDV (ml)	51 ± 38	52 ± 37	− 1.2 ± 7.1	0.98 (0.97–0.99)	0.98	12
LA ESV (ml)	100 ± 39	102 ± 39	− 2.4 ± 10	0.97 (0.93–0.98)	0.97	8.2
LA EF (%)	52 ± 15	51 ± 15	0.50 ± 4.3	0.96 (0.93–0.98)	0.96	6.7
LA GLS (%)	33 ± 13	32 ± 13	0.75 ± 5.4	0.92 (0.85–0.96)	0.92	13

The mean ± standard deviation are reported

*Auto.* automated, *ICC* intra-class correlation coefficient, *CI* confidence interval, *R* correlation value, *CoV* coefficient of variation

**Table 4:** Manual inter-observer agreement ($\mathrm{n}=37$) of left atrial (LA) derived parameters for end-diastolic volume (EDV), end-systolic volume (ESV), ejection fraction (EF) and global longitudinal strain (GLS)

	Manual measures	SO measures	Error measures	ICC (95% CI)	R	CoV (%)
LA EDV (ml)	51 ± 38	51 ± 37	0.14 ± 4.8	0.99 (0.99–1.00)	0.99	9.1
LA ESV (ml)	100 ± 39	97 ± 38	3.2 ± 8.7	0.97 (0.95–0.99)	0.98	7.4
LA EF (%)	52 ± 15	50 ± 15	1.8 ± 4.3	0.95 (0.91–0.98)	0.96	9.5
LA GLS (%)	33 ± 13	34 ± 14	− 0.60 ± 3.8	0.96 (0.93–0.98)	0.96	9.4

The mean ± standard deviation are reported

*SO* second observer, *ICC* intra-class correlation coefficient, *CI* confidence interval, *R* correlation value, *CoV* coefficient of variation

## Results

The automated LA segmentation process was evaluated for accuracy, regarding both segmentation accuracy and accuracy of the clinical metrics, using statistical analysis. The total segmentation processing time in both chambers took 20 s per patient, compared with about 40 min from a manual segmentation.

### Case study of an automated LA segmentation

An example of an automated LA segmentation in end-diastole, mid-systole, end-systole, and mid-diastole, in both views, is illustrated in Fig. 3a, showing excellent agreement with the corresponding manual segmentation. The LA volume and LA strain plots throughout the normalized cardiac cycle of the same subject compare the expert and automated values, as shown in Fig. 3b. This case presents the typical appearance of the LAA and PV ostia in the acquired images and their exclusion from the segmentation. A movie is available as additional file illustrating typical segmentation results across a cardiac cycle with time-resolved volume and strain curves (see Additional file 1).

### Segmentation accuracy

The DSC, MCD, and HD between manual and automated segmentation in all the evaluation set are reported in Table 1. The automated segmentation method achieved an excellent DSC of 0.96 and 0.95, an excellent MCD of 1.2 and 1.3 mm, and a good HD of 5.5 and 6.4 mm, for 2ch and 4ch, respectively. The inter-observer agreement for manual segmentation, reported in Table 2, achieved an excellent DSC of 0.95 and 0.95, an excellent MCD of 1.4 and 1.2 mm, and a good HD of 5.0 and 4.8, for 2ch and 4ch, respectively.

### Clinical metric accuracy

The accuracy of clinical metrics for the LA ESV, LA EDV, LA EF, and LA GLS of the automated method are shown in Table 3 compared against the manual metrics with excellent ICC and low bias. The inter-observer agreement is shown in Table 4. The regression and Bland-Altman plots for the LA parameters between the automated and manual measurements are presented in Fig. 4a, where an excellent correlation and good agreement were observed for each of the four parameters, with a lower accuracy for LA GLS. Figure 4b shows the corresponding inter-observer variability of these metrics. All reported correlation values are significant (p<0.0001).

### Reproducibility due to variability in MV point placement

The reproducibility was studied through the evaluation of the MV points, obtaining the following errors (bias ± SD) on the clinical metrics: 0.31 ± 2.26 ml, − 0.84 ± 5.32 ml, − 0.52 ± 3.24%, and − 0.45 ± 3.43%, for the relative errors of LA EDV, LA ESV, LA EF, and LA GLS, respectively. This variation contributed an insignificant impact on the segmentation accuracy, which yielded a DSC of 0.97 ± 0.02, an MCD of 0.54 ± 0.44 mm, and an HD of 2.18 ± 2.19 mm, compared against the first automated results.

The agreement on the LA segmentation results between the initialization by the semi-automated [22] and the automated [23] method to track the MV points on the clinical metrics was: 0.52 ± 3.89 ml, 0.87 ± 5.06 ml, − 0.13 ± 2.85%, and − 0.03 ± 2.94% for LA EDV, LA ESV, LA EF, and LA GLS, respectively. The MV point placement presented minimal impact on the segmentation accuracy, yielding a DSC of 0.97 ± 0.02, an MCD of 0.48 ± 0.49 mm, and an HD of 2.08 ± 2.19 mm.

### Comparison with an automated LA segmentation method

$\text {CVI}_\text {42}$ produced a clinical-metric error (bias ± SD) against manual measures of 7.83 ± 10.72 ml, 11.07 ± 14.06 ml, and − 3.36 ± 9.19%, with an agreement (ICC) of 0.95, 0.91, and 0.80 for LA EDV, LA ESV, and LA EF, respectively. Although this learning-based method performance is strong, it was outperformed by our proposed method, as seen in Table 3. However, we were only able to compare LA volumes, not segmentations, and LA volumes depend strongly on the evaluation of LA length (also automatically calculated by $\text {CVI}_\text {42}$), which may be a source of discordance.

## Discussion

In this study, a fast, automated method for time-resolved segmentation of the LA in 2ch and 4ch views from standard long-axis cine images was developed and evaluated. The measured values for LA volumes and EF were in agreement with other studies [33]. The proposed method showed excellent agreement with manual segmentation by expert readers. The method extracts clinically relevant LA functional parameters with precision approaching that of expert readers, 120 times faster, and with minimal sensitivity to user input error. This enables accurate, fast, and reproducible assessment of LA volumes and function in clinical routine. Although the approach requiring manual MV point insertion is not fully automated, we developed an automated MV tracking method for obtaining these points using an in-house deep learning-based network [23]. We tested its use in this LA segmentation method, demonstrating that a fully-automated LA segmentation method provides the same accuracy as the semi-automated version. Indeed, the accuracy of this LA segmentation method is not sensitive to variability in the MV points initialization.

While this paper mainly evaluates LA systolic parameters, the results should be similar for diastolic time-points. The proposed method is able to improve the value of cardiac MRI in clinical practice as it allows a fast assessment of phasic LA function. Indeed, LA function, especially in diastole, is rarely evaluated in cardiac MRI clinical routine. As shown in Fig. 4a, the method provides low bias for volumetric values (LA EDV and LA ESV), and functional metrics (LA EF and LA GLS). The slight overestimation in volumes is mainly caused by (i) the exact, automatic delimitation in the LA edge, bigger by one or two-layer pixels than the manual segmentation as its delimitation is observer-dependent, and by (ii) the difference in excluding the PV or LAA, which also influenced a higher HD. The most significant CoV is for LA strain, which is more sensitive than volume due to errors in perimeter delineation, especially with the valve positioning. However, the automatic strain measurement might be better and more reproducible, since manual contouring suffers from inconsistencies between time frames. Despite these differences, the CoVs of these clinical metrics are on par with other reported variations, with a range between 5.7 and 28% [11, 34, 35].

The automated method compared well to other segmentation techniques. For instance, an active contour LA segmentation method which automatically initializes a seed region with the Hough transform technique was evaluated for volumetric 3D MRI of the LA, with a good DSC of 0.82 ± 0.06 (n = 12) [36]. Also applied to 3D volumetric MRI, a multi-view convolutional neural network architecture with an adaptive fusion strategy yielded an excellent DSC of 0.95 (n = 20) [37]. Furthermore, the methods presented in a benchmark study [18] achieved similar performance. However, these methods were targeted to single time frame volumetric imaging of the LA, which is an entirely different application, and if they were applied to cine images, they would not delimit the segmentation with the PV, LAA and the MV boundaries. Other methods, similar to ours, sought to segment the LA in long-axis cine images, as noted above. These include a convolutional-neural-network method with the unscented Kalman filter which yielded an excellent DSC of 0.94 ± 0.04 for 2ch and 0.94 ± 0.08 for 4ch (n = 20) [20], and a similar method with a VGG-16 framework which also yielded an excellent DSC of 0.93 ± 0.05 for 2ch and 0.95 ± 0.02 for 4ch (n = 600) [21]. However, none of these methods reported clinical metrics nor were evaluated in a clinical population. We evaluated commercially available clinical cardiac MRI software capable of automated LA volume derivation ($\text {CVI}_\text {42}$) on our data sets, and found high performance, although it was still lower than our proposed method and unable to derive LA strain values.

The segmentation accuracy of the automated method was similar to the manual inter-observer accuracy, as shown in Fig. 4b. The inter-observer variability displayed a lower standard deviation but higher bias, which is inherent to each observer. The accuracy in the 4ch view was slightly lower than the inter-observer accuracy, meaning the 4ch view is more challenging to segment by this method. However, the automated method segmented the LA in the same manner in every frame, which improves the segmentation consistency along the cardiac cycle.

In the proposed workflow, the edge detection task was a key step prior the edge reconstruction. Within the vast range of edge detection techniques [38], in this application the chosen Canny’s technique achieved a clearer cost image preserving the physiological edges while reducing the imaging artifact noise, compared to Sobel, Prewitt and Roberts edge detection techniques, which were less computationally expensive but achieved a lower overall performance. Future work on this matter would involve the use of an edge detection technique and an adapted problem optimization in a three dimensional space, i.e., a stack of all cine frames.

### Limitations

The main limitation of this study is the small sample size, which did not permit an analysis of how specific imaging issues, such as off-resonance artifacts within the LA blood pool or non-standard slice-prescription, affected segmentation accuracy. Another limitation might be a lack of healthy subjects, since our cohort consisted of mainly AF patients. However, this represents the types of patients who would require LA function evaluation. In our experience, analysis of patients is generally more demanding compared to controls.

Another limitation of the method is the need of initialization with the MV points, although this could be automated with residual neural networks [23]. An initialization task is commonly employed in segmentation problems, e.g. to initiate a thresholding task [39] or a region-growing process [40]. We showed that the inter-observer variability of MV placement, including the automated approach we have developed, minimally impacted the segmentation process.

Another limitation of this work is that total segmentation time which, while a matter of only seconds, was still longer than learning-based methods [20, 21] which require less than a second. Although this difference in time is a pitfall, compared to human labor our method is valuable and an attractive option.

## Conclusion

The developed automated method performs time-resolved segmentation of the LA in cardiac MRI cine images using active contours in a polar grid. The method performs well in assessing LA volumes and strains against manual measurements in a patient population. Furthermore, the method yielded clinical metrics in line with inter-observer variability between expert readers.

Our LA segmentation method, based on active contours automatically initialized by mitral annular points placement, may be introduced as a accurate, fast, and reproducible method for measuring phasic LA volume and strain in cardiac MRI.

## Data Availability

The implemented software will be made freely available to researchers provided that they properly cite the current paper describing the method. The data sets used and/or analysed during the current study available from the corresponding author on reasonable request.

## Abbreviations

2ch: 2-Chamber
4ch: 4-Chamber
AF: Atrial fibrillation
CoV: Coefficient of variation
DSC: Dice similarity coefficient
EDV: End-diastolic volume
EF: Ejection fraction
ESV: End-systolic volume
GLS: Global longitudinal strain
HD: Hausdorff distance
ICC: Intra-class correlation coefficient
LA: Left atrium
LAA: Left atrial appendage
LV: Left ventricle
MA: Mitral annular
MCD: Mean contour distance
MRI: Magnetic resonance imaging
PV: Pulmonary vein
R: Correlation value
